# feedr and animalnexus.ca: A paired R package and user‐friendly Web application for transforming and visualizing animal movement data from static stations

**DOI:** 10.1002/ece3.3240

**Published:** 2017-08-30

**Authors:** Stefanie E. LaZerte, Matthew W. Reudink, Ken A. Otter, Jackson Kusack, Jacob M. Bailey, Austin Woolverton, Mark Paetkau, Adriaan de Jong, David J. Hill

**Affiliations:** ^1^ Department of Geography and Environmental Studies Thompson Rivers University Kamloops BC Canada; ^2^ Natural Resources and Environmental Studies University of Northern British Columbia Prince George BC Canada; ^3^ Department of Biological Sciences Thompson Rivers University Kamloops BC Canada; ^4^ Department of Physics Thompson Rivers University Kamloops BC Canada; ^5^ Department of Wildlife, Fish, and Environmental Studies Swedish University of Agricultural Sciences Umeå Sweden

**Keywords:** feedr, movement, open‐source, R, radio frequency identification, Shiny, user‐friendly, visualization, Web application

## Abstract

Radio frequency identification (RFID) provides a simple and inexpensive approach for examining the movements of tagged animals, which can provide information on species behavior and ecology, such as habitat/resource use and social interactions. In addition, tracking animal movements is appealing to naturalists, citizen scientists, and the general public and thus represents a tool for public engagement in science and science education. Although a useful tool, the large amount of data collected using RFID may quickly become overwhelming. Here, we present an R package (feedr) we have developed for loading, transforming, and visualizing time‐stamped, georeferenced data, such as RFID data collected from static logger stations. Using our package, data can be transformed from raw RFID data to visits, presence (regular detections by a logger over time), movements between loggers, displacements, and activity patterns. In addition, we provide several conversion functions to allow users to format data for use in functions from other complementary R packages. Data can also be visualized through static or interactive maps or as animations over time. To increase accessibility, data can be transformed and visualized either through R directly, or through the companion site: http://animalnexus.ca, an online, user‐friendly, R‐based Shiny Web application. This system can be used by professional and citizen scientists alike to view and study animal movements. We have designed this package to be flexible and to be able to handle data collected from other stationary sources (e.g., hair traps, static very high frequency (VHF) telemetry loggers, observations of marked individuals in colonies or staging sites), and we hope this framework will become a meeting point for science, education, and community awareness of the movements of animals. We aim to inspire citizen engagement while simultaneously enabling robust scientific analysis.

## 1 INTRODUCTION

Radio frequency identification (RFID) can provide a simple, inexpensive solution for examining the behavior and movements of animals (Bonter & Bridge, 2011). Bridge and Bonter (2011) are largely responsible for popularizing this technique by developing and sharing plans for a low‐cost RFID logger that could be made simply and easily (http://www.animalmigration.org/RFID/CheapRFID.htm). Fitting animals with inexpensive, lightweight PIT tags and placing RFID loggers on or near feeders or nests results in a simple technique for logging large numbers of visits to a particular location. Further, due to the low cost of PIT tags, they can be deployed on a large number of individuals, quickly resulting in large amounts of data that, while useful, may be overwhelming for many researchers.

Despite these data challenges, RFID technology has been quickly adopted by researchers studying a variety of animal behaviors (e.g., parental care Bartsch, Weiss, & Kipper, 2015; fledging Johnson, Hebert, Napolillo, & Allen, 2013; interspecific social interactions Farine et al., 2015; foraging patterns Bonter, Zuckerberg, Sedgwick, & Hochachka, 2013; pathogen transmission Adelman, Moyers, Farine, & Hawley, 2015). RFID loggers also have the potential to work well in citizen science projects, as many people enjoy helping to maintain habitat such as nest boxes or feeders, and may be interested in understanding the movements of birds or other animals throughout the urban landscape. Citizen science is a powerful tool for investigating urban ecosystems and informing conservation efforts (Cooper, Dickinson, Phillips, & Bonney, 2007). For citizen science projects to succeed, however, participation must be maintained through effective communication with, and regular feedback to, participants (Dickinson et al., 2012). Therefore, any RFID‐based citizen science project would benefit from being able to quickly and easily summarize and provide clear and interesting visualizations of RFID data.

RFID systems are therefore powerful in that they can collect large amounts of data, but difficult in that the amount of data may be overwhelming for many scientists and projects. Further, RFID data require substantial postprocessing and can be challenging to interpret. The technical and computer skills required to process the data also create problems of accessibility, making it difficult for nonspecialists or students to access and interpret the data.

Free and open‐source software (FOSS) is ideal for providing researchers with tools to deal with large amounts of data. The “free” aspect reduces financial barriers to its use, and the “open‐source” aspect permits and encourages collaboration which can result in better, more powerful software. The statistical software environment, R (R Development Core Team, 2008), is FOSS and allows users to develop powerful packages to extend its use. In fact, there are already several R packages which provide valuable tools for analysis of raw or transformed RFID data (e.g., social network analysis in asnipe Farine, 2017; dominance interactions aniDom Farine & Sanchez‐Tojar, 2017; Dominance Fujii et al., 2016; Perc Krueger & Krueger, 2016); however, there are few if any packages that focus on broad data transformations/summarizations and visualizations of RFID data.

R has a major drawback of being challenging to learn, which again creates problems of accessibility. In contrast, other systems that rely purely on easy‐to‐use graphical user interfaces may lack the complexity and flexibility that more advanced users desire. Fortunately, a new R project, Shiny Web applications (apps), has been recently developed to provide a framework for creating user‐friendly, R‐based Web apps (Chang, Cheng, Allaire, Xie, & McPherson, 2016).

By combining traditional R functions with Shiny Web apps, we have developed a free and open‐source tool for addressing the challenges associated with managing RFID (and similar) data. This tool is simultaneously powerful, flexible, and customizable while also being user‐friendly. The R package, feedr, provides the basic tools and functions, while the online Shiny Web app (http://animalnexus.ca) permits users to apply feedr functions to their RFID data through a user‐friendly interface without having to install R or the feedr package. Further, we provide several conversion functions which help users format RFID data for use by other R packages. While our package was initially developed for visualizing movements of small birds carrying PIT tags between RFID loggers mounted on feeders, we have developed feedr with flexibility in mind; it can be used for any data set in which uniquely identified individuals have time‐stamped visits recorded at georeferenced locations. These may include data collected from hair traps, static very high frequency (VHF) telemetry loggers, or even observations of marked individuals in colonies or staging sites.

In addition to accessing feedr tools through a user interface, the animalnexus.ca site provides access to a database of raw RFID data. These data are comprised of contributed data from different projects, species, and locations. A large part of this database is data from our ongoing project at Thompson Rivers University (TRU) in Kamloops, BC, Canada. These data are actively collected via WiFi from a suite of RFID‐enabled feeders spread across campus. A snapshot of current activity is always visible on the animalnexus.ca homepage.

## 2 GENERAL USAGE OF FEEDR

**Figure 1:**
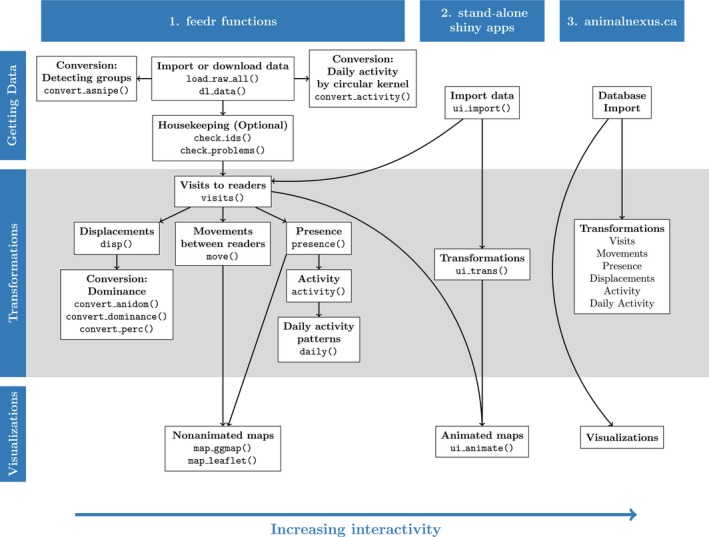
The feedr package can be used in three different (but not mutually exclusive) ways. First, there are the basic package functions to be used in R. These give the user the most control over the data and the output. Second, there are standalone Shiny apps which launch user interfaces from R. This allows users familiar with R to use a combo of the basic R functions and easy‐to‐use interfaces. Finally, by accessing the online Web app at animalnexus.ca, users can load, transform, and visualize data without using R directly. Usage is indicated by functions()or by site location. Arrows indicate work flow order

feedr is an R package with a suite of functions for loading, transforming, and visualizing RFID data. feedr functionality can be accessed in three different ways (Figure 1): (1) R enthusiasts can use feedr functions directly through scripting in R; (2) intermediate users can complement and simplify their scripting in R by launching standalone Shiny apps; (3) nonspecialist users, including students or citizen scientists, can use the publicly accessible animalnexus.ca site to load and manipulate data, access the database, or get up‐to‐date information on the movements of local birds and other project contributions without having to use R at all. Currently, our package handles RFID data collected from loggers that continuously record the presence of an individual. These raw reads can be imported and then transformed to visits, after which they can be transformed to other measures such as movements or presence around a logger.

The following coding examples are designed to be run in the R console after loading the feedr package (library(feedr)). All data sets used here are available for download and are included in the feedr package (see Data Availability). For an example of a complete typical workflow, see the R script in Supporting Information.

### 2.1 Loading data

**Figure 2:**
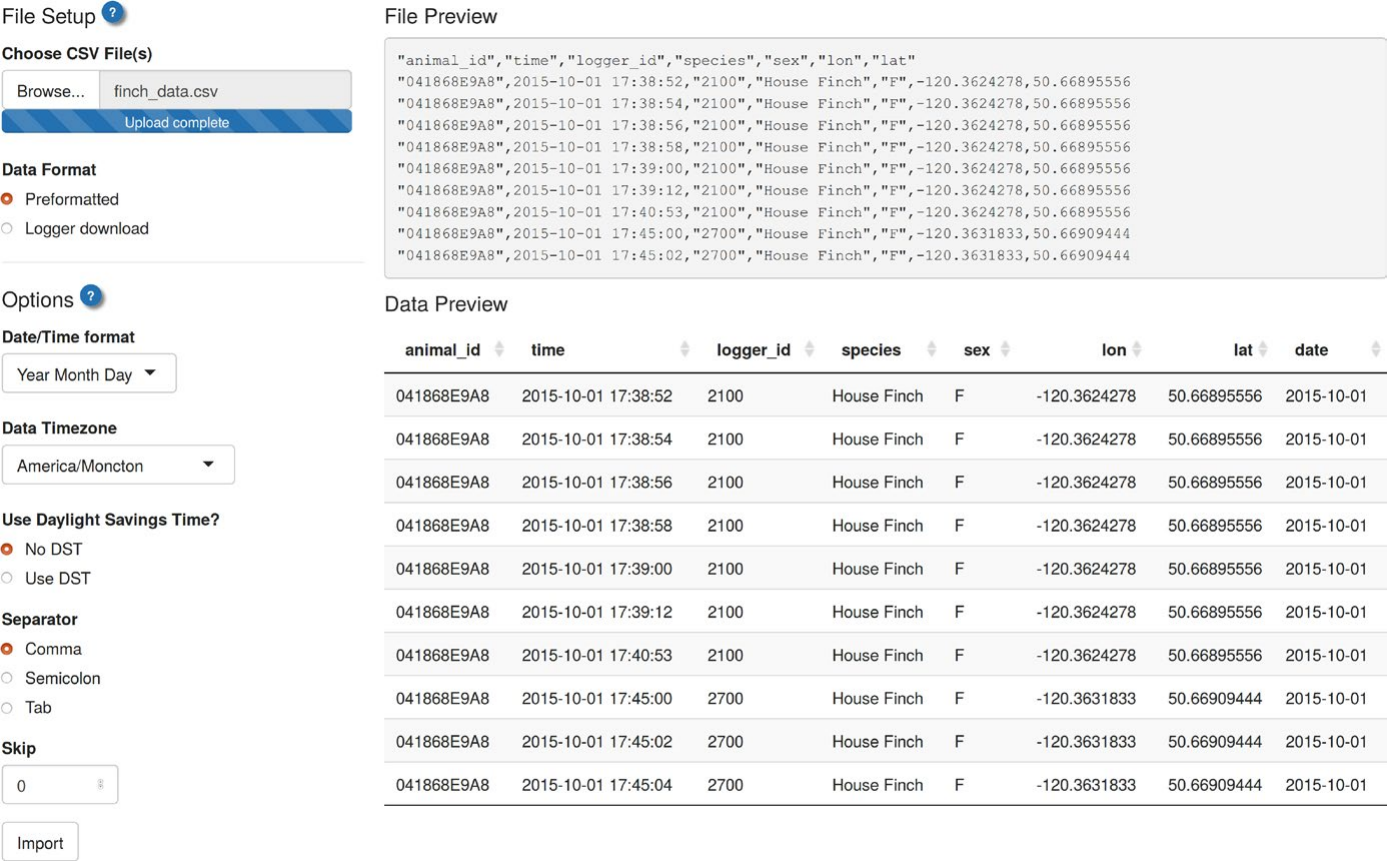
User interface for importing data. Accessible through the “Import” tab at animalnexus.ca or by calling the function ui_import()

Data can be imported through R scripting, through a standalone Shiny app (function ui_import(); Figure 2), or through animalnexus.ca under the “Import” tab (Figure 1 top row). Note that data imported online are only available to the user and is not accessed or stored for future use by the animalnexus.ca project. Alternatively, those who would like to share their data can contribute it to the online animalnexus.ca database by contacting the development team (SEL, DJH or MWR).

Raw data can be loaded directly for use by feedr provide it has the required columns: animal_id, logger_id, and time (lat and lon are also required for visualizations). Users can then format their data with the feedr function load_format().
raw <‐ read.csv(“finch_data.csv”)
raw <‐ load_format(raw)



By default load_format expects the time column to be formatted as Year‐Month‐Day Hour:Min:Sec (e.g. 2017‐05‐21 14:50:43), but different time formats can be specified with the time_format argument which indicates the order of year (y), month (m), day (d), hour (H), min (M), sec (S), and, if necessary AM/PM (p). feedr functions extract date/times using the lubridate package (Grolemund & Wickham, 2011), and thus, the exact format is quite flexible. For example, time formatted as “31‐12‐17 09:12 pm” would be extracted using: 
raw <‐ load_format(raw, time_format = “dmy HM p”)



Data can also be loaded with the load_raw_all() function, which loads and combines all data files in a folder. Here, each data file is expected to correspond to a single RFID logger (there can be more than one data file per logger). Each file should have three *unlabeled* columns corresponding to animal_id, date, and time (time in this case including only hours, minutes, and seconds).
raw_all <‐ load_raw_all(r_dir = “./data‐folder/”)



The details argument specifies where the logger_id can be found. details = 1 (default) indicates that the logger_id is in the first line of each file. For more advanced users, details = 0 extracts logger_id from the file name, and users can supply a regular expression to the logger_pattern argument to extract only parts of the file name (otherwise the entire file name is used). 
raw_all <‐ load_raw_all(r_dir = “./data‐folder/”,
details = 0)



By default, data are expected to be formatted as from TRU RFID‐enabled feeders, but there is enough flexibility to specify formats from other systems. For example, the default is for data columns to be separated by whitespace, but the sep argument can be changed to “,” or “;” to reflect comma‐ or semicolon‐separated data. Similarly to the load_format() function, the combined format of the date and time columns can be specified by changing the time_format argument (defaults to “mdy HMS”). 
raw_all <‐ load_raw_all(r_dir = “./data‐folder/”,
time_format = “dmy HMS”,
sep = “,”)



Although raw logger files do not normally contain logger coordinates, they can be added to the data in several ways. First, specifying details = 2 extracts the logger_id from the first line of each file and also extracts the logger coordinates in the format of lat, lon from the second line of each file. Thus if users add logger coordinates to their files, they will be automatically extracted. 
raw_all <‐ load_raw_all(r_dir = “./data‐folder/”,
details = 2)



Alternatively, the imported data can be merged with a logger index file containing the columns logger_id, lat and lon. 
logger_index <‐ read.csv(“logger_index.csv”)
raw_all <‐ load_raw_all(r_dir = “./data‐folder/”)
raw_all <‐ merge(raw, logger_index,
by = “logger_id”)



**Figure 3:**
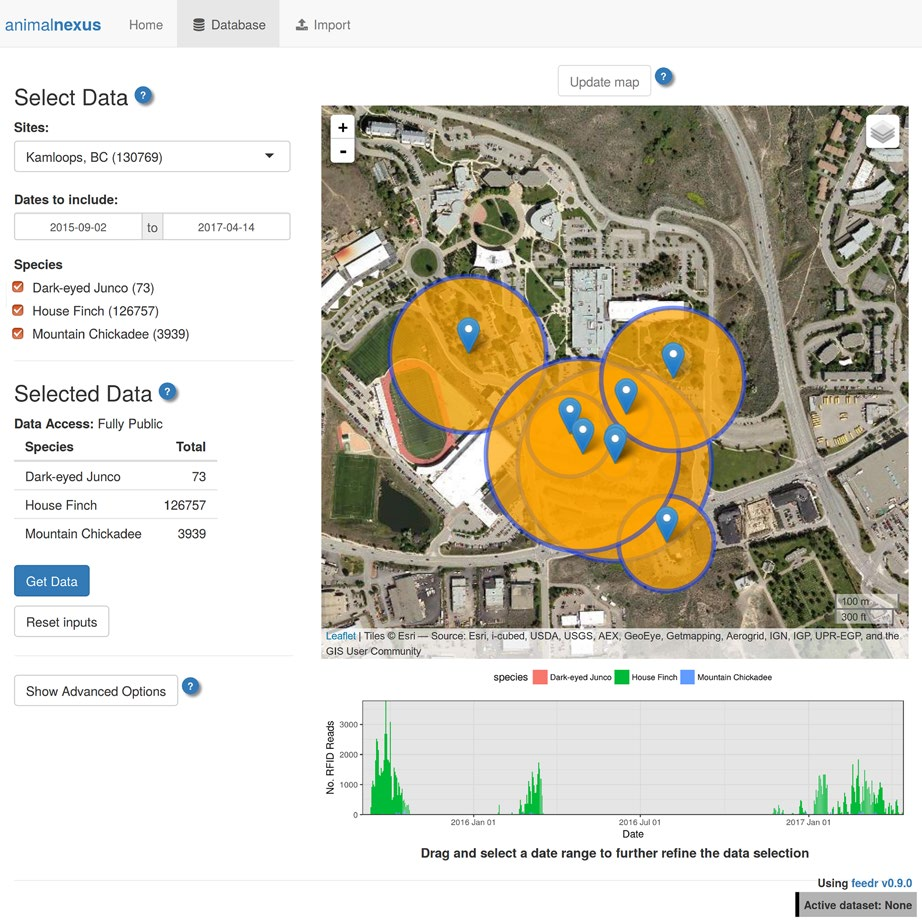
Access to publicly available RFID data from the database hosted at animalnexus.ca. Accessible through the “Database” tab

Those who would like to use the data stored in the online database can either access the data at animalnexus.ca by clicking on the “Database” tab (Figure 3) or can use the dl_data() function:
raw_dl <‐ dl_data(start = “2015‐09‐01”,
end = “2015‐11‐01”)



### 2.2 Transformations

**Figure 4:**
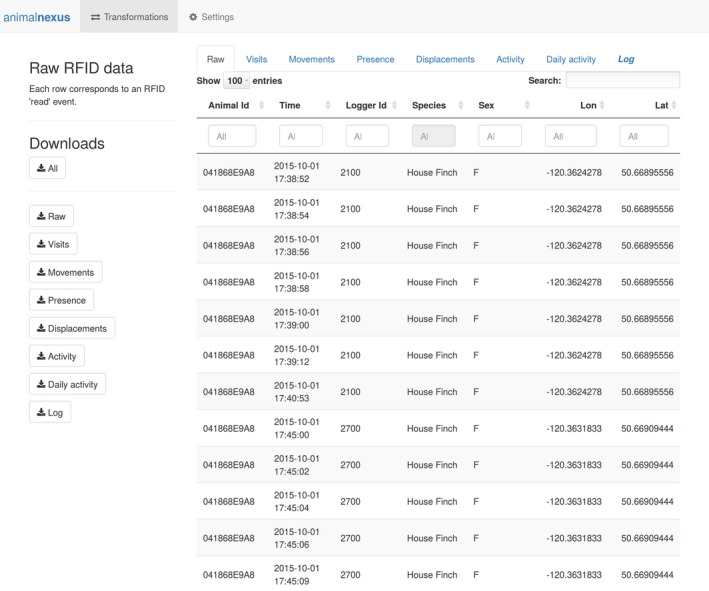
User interface for transforming data. Accessible through the “Transform” tab at animalnexus.ca or by calling the function ui_trans()

Once loaded, RFID data can be converted into other data types using various feedr transformation functions through R scripting, by launching a standalone Shiny app from R (function ui_trans(); Figure 4), or by clicking on the “Transformations” tab on the animalnexus.ca site (Figure 1 middle row).

Most users would first use the visits() function to consolidates scans of individual RFID tags (e.g., Table 1) into logger visits (e.g., Table 2). RFID reads are consolidated if the time between two reads is less than a particular cutoff (defaults to 3 s; specified by the bw argument). In TRU RFID‐enabled feeders, the receiver is embedded in the perch; thus, RFID reads reflect time the individual was at the feeder.
v <‐ visits(r = raw, bw = 4)



**Table 1:** Example of raw house finch RFID data. animal_id refers to the unique PIT tag code logged by an RFID receiver. time is the time at which the PIT tag was detected. logger_id is the unique ID of the RFID logger. species and sex are the species and sex of the animal. lat and lon are the latitude and longitude of the corresponding RFID‐enabled feeder

animal_id	time	logger_id	species	sex	lon	lat	date
041868E9A8	2015‐10‐01 17:38:52	2100	House Finch	F	−120.3624	50.66896	2015‐10‐01
041868E9A8	2015‐10‐01 17:38:54	2100	House Finch	F	−120.3624	50.66896	2015‐10‐01
041868E9A8	2015‐10‐01 17:38:56	2100	House Finch	F	−120.3624	50.66896	2015‐10‐01
041868E9A8	2015‐10‐01 17:38:58	2100	House Finch	F	−120.3624	50.66896	2015‐10‐01
041868E9A8	2015‐10‐01 17:39:00	2100	House Finch	F	−120.3624	50.66896	2015‐10‐01
041868E9A8	2015‐10‐01 17:39:12	2100	House Finch	F	−120.3624	50.66896	2015‐10‐01
041868E9A8	2015‐10‐01 17:40:53	2100	House Finch	F	−120.3624	50.66896	2015‐10‐01
041868E9A8	2015‐10‐01 17:45:00	2700	House Finch	F	−120.3632	50.66909	2015‐10‐01
041868E9A8	2015‐10‐01 17:45:02	2700	House Finch	F	−120.3632	50.66909	2015‐10‐01
041868E9A8	2015‐10‐01 17:45:04	2700	House Finch	F	−120.3632	50.66909	2015‐10‐01

**Table 2:** Example of house finch visit data, data output from the visits() function. These data show multiple reads collapsed into individual visits. animal_id refers to the unique PIT tag code logged by an RFID receiver. date is the day on which the visit was started. start and end are the start and end times of the visit made to an RFID‐enabled feeder. logger_id is the unique ID of the RFID logger. Note that for simplicity, some columns with specific animal or logger information have been omitted

animal_id	date	start	end	logger_id
041868E9A8	2015‐10‐01	2015‐10‐01 17:38:52	2015‐10‐01 17:39:00	2100
041868E9A8	2015‐10‐01	2015‐10‐01 17:39:12	2015‐10‐01 17:39:12	2100
041868E9A8	2015‐10‐01	2015‐10‐01 17:40:53	2015‐10‐01 17:40:53	2100
041868E9A8	2015‐10‐01	2015‐10‐01 17:45:00	2015‐10‐01 17:45:56	2700
041868E9A8	2015‐10‐01	2015‐10‐01 17:46:31	2015‐10‐01 17:46:31	2700
041868E9A8	2015‐10‐01	2015‐10‐01 17:51:03	2015‐10‐01 17:51:16	2700
041868E9A8	2015‐10‐01	2015‐10‐01 17:51:42	2015‐10‐01 17:51:58	2700
041868E9A8	2015‐10‐01	2015‐10‐01 17:52:51	2015‐10‐01 17:53:07	2700
041868E9A8	2015‐10‐01	2015‐10‐01 17:53:41	2015‐10‐01 17:54:57	2700
041868E9A8	2015‐10‐01	2015‐10‐01 17:56:42	2015‐10‐01 17:56:42	2700

Visit data can then be transformed into a variety of different data types (Figure 1 left panel). For example, depending on the behavior of a species and if the logger is part of a feeder, displacement events may occur when one individual is forced away from the feeder by another, presumably more dominant, individual (*cf* Adelman et al., 2015). The disp() function summarizes these events using a time cutoff between successive visits by different individuals (defaults to 2 s; specified by the bw argument). There are many R packages for calculating dominance; thus, we provide several conversion functions to convert the output of the disp() function to formats compatible with these packages. For example, our function convert_anidom() formats data for use by the aniDom package which calculates dominance hierarchies based on Elo scores (Farine & Sanchez‐Tojar, 2017). The convert_dominance() function formats data for the Dominance package which calculates an average dominance index (Krueger & Krueger, 2016). Finally, the convert_perc() function formats data for the Perc package, which calculates dominance from networks based on percolation and conductance (Fujii et al., 2016). 
d <‐ disp(v, bw = 1)
i <‐ convert_anidom(d)
i <‐ convert_dominance(d)
i <‐ convert_perc(d)



Individual movements can be tracked between loggers (move(); Table 3), or each individual's visits to a specific logger can be lumped together into a measure of “presence” (presence(); Table 4). Presence reflects the amount of time an individual spent making regular visits to a logger. Visits are considered regular if the time between them was less than a particular cutoff (defaults to 15 min; specified by the bw argument). Presence contrasts with visits, which reflect the amount of time actually spent in range of a logger (if a feeder, presumably on the perch). 
m <‐ move(v)
p <‐ presence(v, bw = 20)



**Table 3:** Example of house finch movements between loggers, data output from the move() function. Each movement is described by two rows: when the individual left a logger and when it arrived at a new logger. animal_id refers to the unique PIT tag code logged by an RFID receiver. date is the day on which the event (either arriving or leaving) was made. time is the time of the specific event (arrived/left). logger_id is the unique ID of the RFID logger the bird arrived at or left from. direction is the direction of the movement with respect to the particular RFID logger (arrived/left). move_id identifies specific movement events for a particular individual. move_dir is a category specifying to and from which RFID loggers the individual was moving. move_path is a category specifying between which RFID loggers the individual was moving (independent of direction). strength reflects the connectivity between loggers and is the inverse of the time it took to arrive at a logger after having left the previous logger. Note that for simplicity, some columns with specific bird or logger information have been omitted

animal_id	date	time	logger_id	direction	move_id	move_dir	move_path	strength
041868E9A8	2015‐10‐01	2015‐10‐01 17:40:53	2100	Left	1	2100_2700	2100_2700	14.57
041868E9A8	2015‐10‐01	2015‐10‐01 17:45:00	2700	Arrived	1	2100_2700	2100_2700	14.57
041868E9A8	2015‐10‐01	2015‐10‐01 17:56:42	2700	Left	2	2700_2200	2200_2700	0.06
041868E9A8	2015‐10‐02	2015‐10‐02 10:57:00	2200	Arrived	2	2700_2200	2200_2700	0.06
041868E9A8	2015‐10‐06	2015‐10‐06 14:24:50	2200	Left	3	2200_2700	2200_2700	0.60
041868E9A8	2015‐10‐06	2015‐10‐06 16:05:40	2700	Arrived	3	2200_2700	2200_2700	0.60

**Table 4:** Finch presence at different loggers, data output from the presence() function. animal_id refers to the unique PIT tag code logged by an RFID receiver. date is the day on which the start of the presence bout was recorded. logger_id is the unique ID of the RFID logger the bird arrived at or left from. start and end are the start and end times of the period of presence. length is the length of the period in minutes (end time minus start time). Note that for simplicity, some columns with specific bird or logger information have been omitted

animal_id	date	logger_id	start	end	length
041868E9A8	2015‐10‐01	2100	2015‐10‐01 17:38:52	2015‐10‐01 17:40:53	2.016667 mins
041868E9A8	2015‐10‐01	2700	2015‐10‐01 17:45:00	2015‐10‐01 17:56:42	11.700000 mins
041868E9A8	2015‐10‐02	2200	2015‐10‐02 10:57:00	2015‐10‐02 11:11:43	14.716667 mins
041868E9A8	2015‐10‐02	2200	2015‐10‐02 12:45:37	2015‐10‐02 13:06:27	20.833333 mins
041868E9A8	2015‐10‐02	2200	2015‐10‐02 14:22:58	2015‐10‐02 14:27:30	4.533333 mins

Note that move() and presence() are calculated individually without reference to other animals that may also be present. For users who wish to infer group membership by examining covisits to a logger, we provide the convert_asnipe() function which formats loaded data for use by the gmmevents() and get_associations_points_tw() functions in the asnipe package (Farine, 2017). These functions allow users to assign group membership by either applying Gaussian mixture models or using a time window approach, respectively. 
i <‐ convert_asnipe(r, fun = “gmmevents”)
i <‐ convert_asnipe(r, fun =
“get_associations_points_tw”)



Finally, activity patterns can also be inferred by summarizing the times at which individuals’ presence is detected by the logger. The activity() function transforms presence data into binary active/inactive data scored for given time intervals (defaults to a resolution of 15 min; specified by the res argument). This binary activity can then be summarized into average daily (24‐h) activity patterns using the daily() function. 
a <‐ activity(p, res = 5)
da <‐ daily(a)



Alternatively, users can use the convert_activity() function to create a list of time values suitable for use in the fitact() function from the activity package which calculates average daily activity patterns using circular kernel probability density functions.

### 2.3 Visualizations

feedr also provides tools for visually summarizing the time present at, and movements between, loggers. As with transformations, visualizations can be constructed using the feedr functions directly through R scripting, by launching the standalone Shiny app from R (function ui_animate(); see below), or through the animalnexus.ca site under the “Visualizations” tab (Figure 1 bottom row).

Visualizations created using the base feedr package functions via R scripting are more customizable and allow users to create static maps using map_ggmap() (i.e., images; through the ggmap package; Kahle & Wickham, 2013) or interactive maps using map_leaflet() (i.e., maps that can be panned and zoomed; through the leaflet package; Cheng & Xie, 2016).

Visualizations always represent summarized data and users can specify one of three built in summaries. The first, summary = “sum”, simply expresses the total time spent around a logger and the total number of movements made between loggers. 
map_ggmap(p = p, m = m, summary = “sum”)
map_leaflet(p = p, m = m, summary = “sum”)



**Figure 5:**
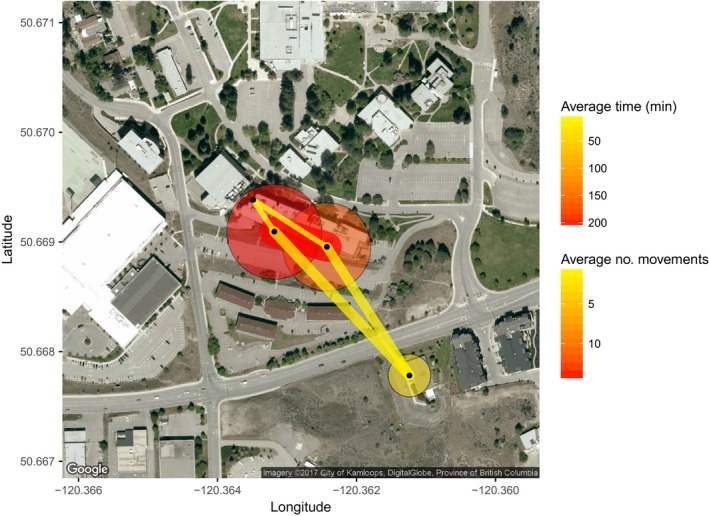
Static map of presence at, and movements between, loggers summarized across 11 individuals over 42 days. Circles represent total time present at each RFID logger (small yellow = less time; large red = more time), and lines represent path use (narrow yellow = less use; wide red = more use). Black circles indicate logger locations. Created using the map_ggmap() function

**Figure 6:**
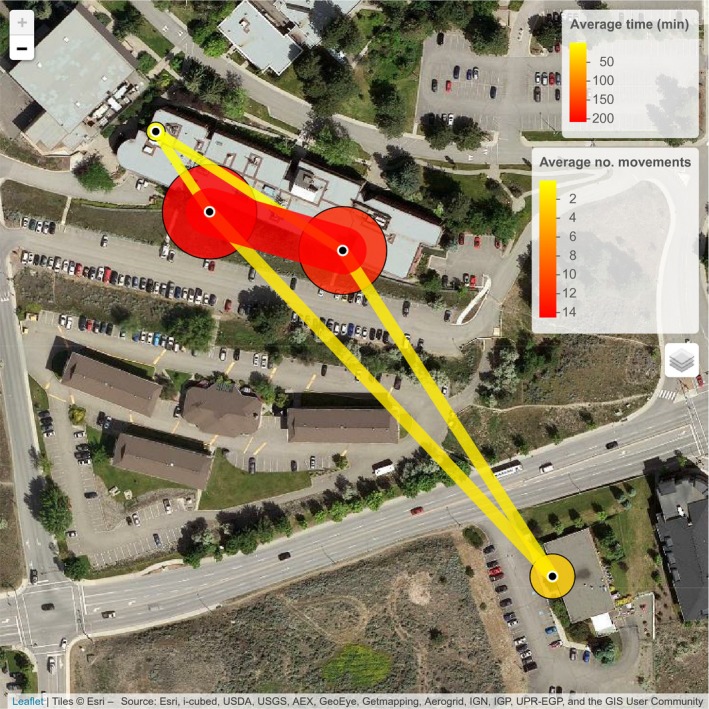
Interactive map of presence at and movements between loggers summarized across 11 individuals over 42 days. Circles represent total time present at each logger (small yellow = less time; large red = more time), and lines represent path use (narrow yellow = less use; wide red = more use). White‐outlined black circles represent logger locations. Created using the map_leaflet()

The second, summary = “sum_indiv”, calculates total time and total movements, but averages by the number of individuals. Note that other arguments, such as legend titles, can also be specified (static map Figure 5; interactive map Figure 6).
map_ggmap(p = p, m = m, summary = “sum_indiv”,
p_title = “Average Time (min)”,
m_title = “Average no. movements”)
map_leaflet(p = p, m = m, summary = “sum_indiv”,
p_title = “Average Time (min)”,
m_title = “Average no. movements”)



**Figure 7:**
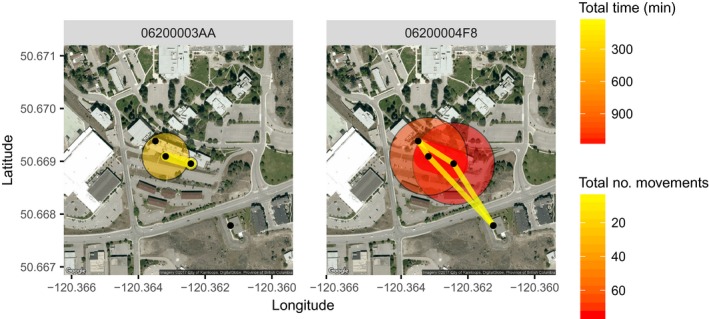
Static map of presence at and movements between loggers summarized for two individuals over 26 and 42 days, respectively. Circles represent total time present at each logger (small yellow = less time; large red = more time), and lines represent path use (narrow yellow = less use; wide red = more use). Black dots indicate logger locations. Created using the map_ggmap() function

Alternatively, visual summaries can be created for specific individuals with map_ggmap using summary = “indiv” and by specifying animal_id's in the which argument (Figure 7):
map_ggmap(p = p, m = m, summary = “indiv”,
p_scale = 0.75, #tweak scaling
p_title = “Total Time (min)”,
m_title = “Total no. movements”,
which = c(“06200003AA”, “06200004F8”))



Users wanting more control over summary types can also summarize their data themselves and use the summary = “none” option (see the next example for more details).

**Figure 8:**
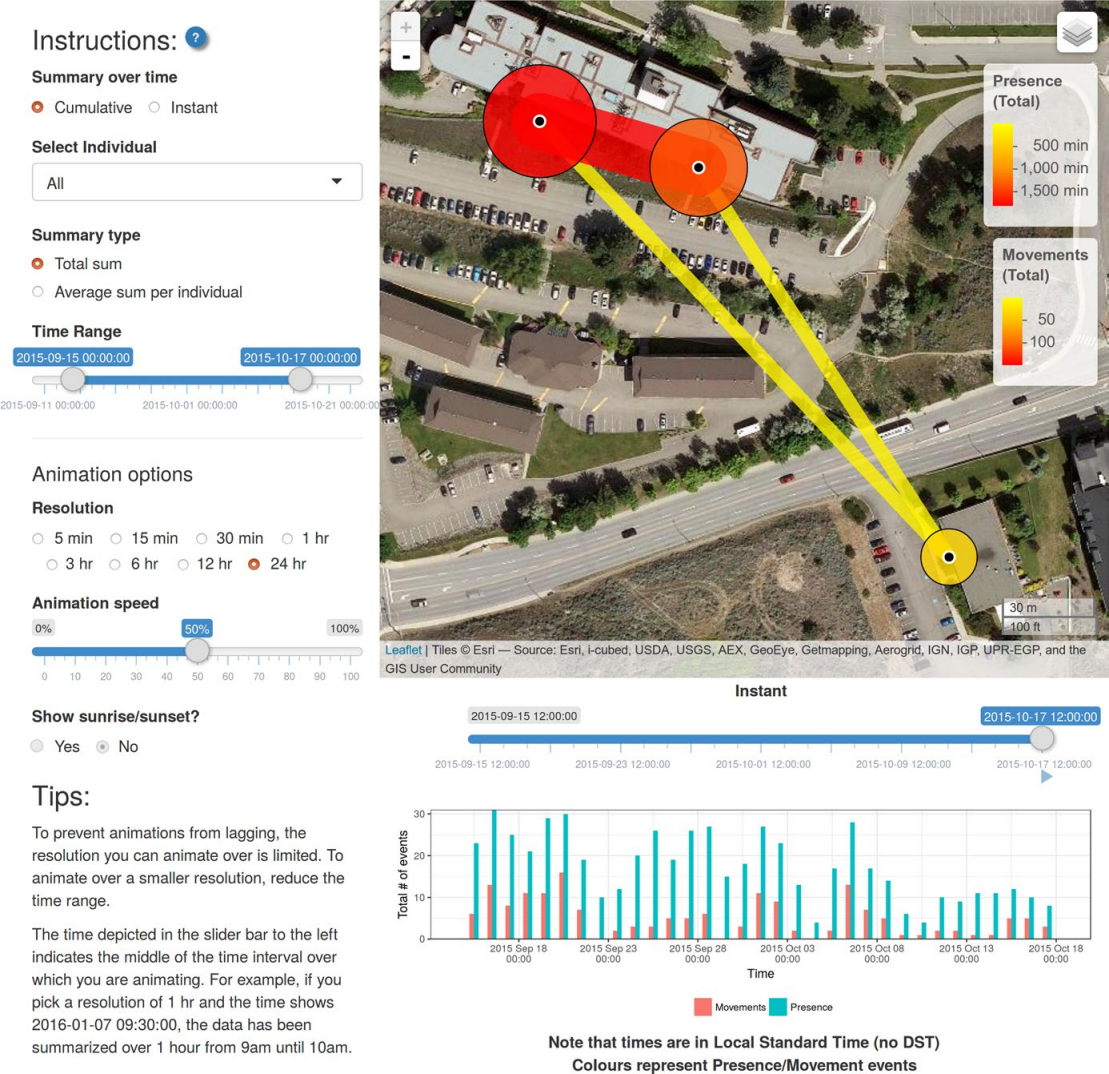
User interface for creating animations of presence at and movements between loggers over time. Accessible through the ui_animate() function or through animalnexus.ca under the “Visualizations” tab

Finally, simple visual animations can also be created. These can either be created through animalnexus.ca under the “Visualizations” tab, or through a standalone Shiny Web app launched with the R function ui_animate(). This function will launch a local Shiny app which allows the user to specify the details of the animation (e.g., cumulative vs. instantaneous, all individuals vs. specific individuals, type of summary; Figure 8).

### 2.4 An advanced example of a scientific application: Gap‐crossing

In order to assess gap‐crossing in black‐capped chickadees (*Poecile atricapillus*), Bailey et al. (2016) used four RFID‐enabled feeders set out in a square pattern surrounding a man‐made gap in the forest (e.g., road, powerline). Loggers recorded visits by PIT‐tagged chickadees for 2 weeks, after which the setup was moved to a new site and the experiment repeated. Thus, Bailey et al. (2016) monitored the time spent at, and movements between, the different feeders. Movements were then compared within and among sites, while assessing the presence/absence of a gap between two feeders, gap distances, and gap type or habitat type.

This is a more advanced example of RFID data manipulation because the preliminary data collected from two sites in this experiment need to be kept separate. As the experiments were located in nearby sites, some individuals participated in both experiments, but we do not want to consider these movements between experiments. We could have separated the data and performed transformations on each subset by hand, but using summarizing functions from the dplyr R package we are able to simultaneously transform each experiment separately (Wickham & Francois, 2016).

Here, we use the group_by() function to first group the data according to the column experiment, and we then apply the transformation functions to each data grouping using the do() function. The %>% is a pipe that sends output from one line to the next line, and the period (.) reflects where the input should go in the do() function. We use ungroup() at the end to remove any remaining grouping. Essentially, the following code loads the data, groups by experiment, and transforms into visits, followed by presence and then movements. 
library(dplyr)
chickadees <‐ read.csv(“chickadee_data.csv”)
chickadees <‐ load_format(chickadees,
tz = “America/Vancouver”)
# Transform the raw data into visit data
# separately for each experiment (site)
v <‐ chickadees %>%
group_by(experiment) %>%
do(visits(.)) %>%
ungroup()
# Transform the visit data into movements and
# presence data
p <‐ v %>%
group_by(experiment) %>%
do(presence(.))%>%
ungroup()
m <‐ v %>%
group_by(experiment) %>%
do(move(.))%>%
ungroup()



Next, in order to maintain the separation of experiments, we summarize the data “by hand” using the summarize() function, also from the dplyr package. To summarize the total sum of presence at each logger in each experiment, we first group by experiment as well as by logger_id (including logger specific information such as lat and lon). To calculate the total number of movements made between each logger, we also group by move_path. Each total is averaged by the number of individuals, calculated previously by the visits() function as animal_n. 
# Average time present per bird, per experiment
p_avg <‐ p %>%
group_by(experiment, logger_id, lat, lon) %>%
summarize(amount = sum(length) / animal_n[1]) %>%
ungroup()
# Path use standardized by the number of birds,
# per experiment
m_avg <‐ m %>%
group_by(experiment, move_path, logger_id, lat, lon) %>%
summarize(path_use = length(move_path) / animal_n[1]) %>%
ungroup()



**Figure 9:**
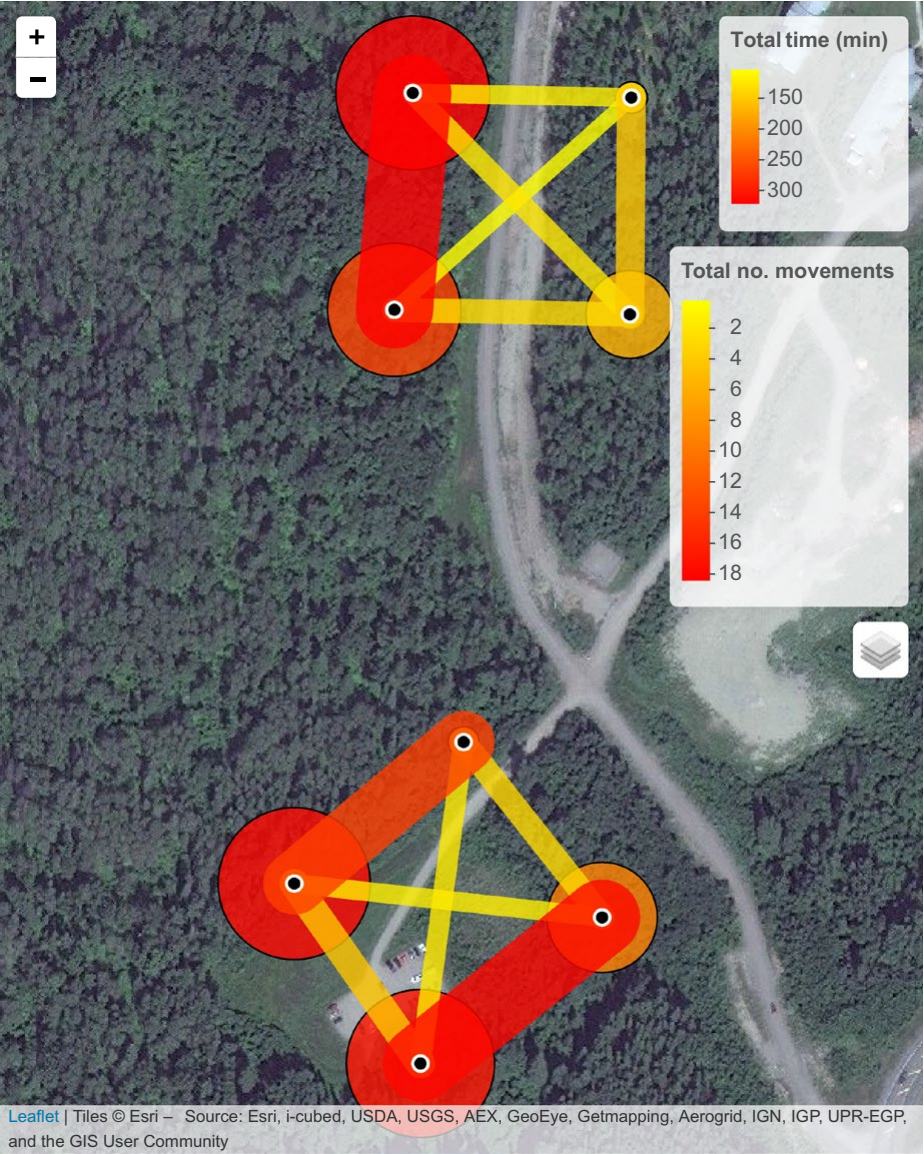
Gap‐crossing in black‐capped chickadees (*Poecile atricapillus*) measured by movements between RFID‐enabled feeders and visualized using the map_leaflet() function from the feedr package. Data are summarized across 20 and 16 individuals over 14 days in each experiment (site), respectively

Now, we are able to plot the data together on the same map, but without showing any movements between experiments (Figure 9).
map_leaflet(p = p_avg, m = m_avg,
summary = “none”,
p_title = “Average Time (min)”,
m_title = “Average no. movements”)



As such, using the feedr package in conjunction with the dplyr package we are able to perform fairly complex data transformations and summaries, and then visualize the results of these experiments.

### 2.5 Other applications: citizen science and education

In addition to numerous other scientific applications, the animalnexus.ca site and database can also be used to facilitate citizen science and/or provide educational opportunities for participants and students. At TRU in Kamloops, BC, we are expanding our network of RFID‐enabled feeders into residential areas as part of a citizen science project examining the impacts of urbanization on avian behavior and ecology. All data will be automatically added to the openly available database on animalnexus.ca. It is our hope that this project will engage the interest of community members.

We are also adding contributions from other projects covering different species from different locations to our database. As a result, this database will become a powerful teaching tool. It is often suggested that students are more engaged in science when they feel that their work is connected to the “real world” and when they are actively participating in science through inquiry‐based learning (Barron & Darling‐Hammond, 2008; Jenkins, 2011). By working on animalnexus.ca, student engagement can be improved by having students work with real data that has not been carefully curated or processed to provide preconceived answers.

## 3 FUTURE DIRECTIONS

Currently, the feedr package can be used for transformations and visualizations. However, one of the powerful aspects of FOSS is the ability to continually add and integrate functionality, such as community contributions as well as analyses from existing and future R packages. Based on user input, we plan to add more types of transformations to the package and more customizations to the animalnexus.ca site. We also plan to include the use of other RFID data formats, such as systems that have start and end times of visit already calculated, or nest boxes that record visits as in or out.

## 4 PACKAGE AVAILABILITY

As of writing, feedr v0.9.0 is available for download on github and can be downloaded and installed directly in R using the devtools package: 
install.packages(“devtools”)
devtools::install_github(“animalnexus/feedr”)



For more specific installation details or for troubleshooting, please see the README (http://github.com/animalnexus/feedr). More specific and technical tutorials can be found at https://animalnexus.github.io/feedr/.

## 5 DATA AVAILABILITY

All data used to illustrate feedr are available in Supporting Information. In addition, the chickadee and finch data sets are part of the feedr package, accessible by calling finches_lg, or chickadees from the R console.

## CONFLICT OF INTEREST

None declared.
